# Analysis of Internal Quality Changes in Apples During Storage Using Near-Infrared Spectroscopy

**DOI:** 10.3390/foods14081412

**Published:** 2025-04-18

**Authors:** Yande Liu, Siwei Lv, Xiaogang Jiang, Yeqing Lu, Bo Hu

**Affiliations:** 1 Institute of Intelligent Mechanical and Electrical Equipment Innovation, East China Jiaotong University, Nanchang 330013, China; 17376597339@163.com (S.L.); jxg_ecjtu@163.com (X.J.); 2 Anhui VSEE Optoelectronic Technology Co., Ltd., Hefei 230093, China; vision-colorsorter@hotmail.com (Y.L.); admin@visionsort.cn (B.H.)

**Keywords:** near-infrared spectroscopy, dynamic online, different varieties of apples, storage period

## Abstract

This study aims to comprehensively evaluate the internal quality changes in apples during storage via near-infrared spectroscopy. Specifically, we focus on the performance differences in different apple varieties under diverse storage conditions and construct predictive models to determine the optimal storage period. By using near-infrared spectroscopy technology, 384 samples of four apple varieties (Xinjiang Akesu, Wafangdian Huangyuanshuai, Shandong Fuji, and Luochuan Fuji) were analyzed to monitor the changes in their soluble solid content (SSC) and fruit firmness within 7 weeks. The results indicated that, under cold storage conditions, SSC and firmness gradually decreased after peaking between the third and fifth weeks, while the opposite trend was observed at room temperature. To enhance the predictive accuracy of the model, several pretreatment methods were employed, including standardization, multiplicative scatter correction (MSC), and standard normal variate transformation (SNV). Additionally, competitive adaptive reweighted sampling (CARS) and uninformative variable elimination (UVE) were utilized for band selection. These pretreatment and selection processes significantly reduced noise and improved model reliability. The best results were achieved with the Normalization-CARS-PLS model for the sugar content at 1 °C, which demonstrated an optimal predictive correlation coefficient (Rp) of 0.904 and a root mean square error of prediction (RMSEP) of 0.67. For firmness at room temperature, the Normalization-CARS-PLS model also showed an excellent performance, with an Rp of 0.823 and an RMSEP of 0.809. The study of the quality of four varieties of apples under three storage conditions in this paper was able to analyze the changes in the internal quality of apples and predict the optimal storage period of different varieties of apples, which is important for guiding the optimal storage period of apples before ripening.

## 1. Introduction

Apples are the third most cultivated and consumed fruit in the world, following bananas and watermelons [1]. Consumers have high demands for their quality, making it essential to study their internal quality, which can be impacted by different storage times and methods. SSC and firmness are two important indicators for evaluating the internal quality of apples, directly influencing their taste and flavor, and thereby affecting consumer acceptance [2]. As apples mature from July to early November, their quality from May to June is particularly important for annual sales. The analysis of spectral changes from May to June is of great significance for apple research. During this period, significant changes occur in internal quality indicators of apples, such as SSC and fruit firmness. Real-time monitoring of these changes using near-infrared spectroscopy helps growers assess apple quality in a timely manner and optimize the harvest timing. Different apple varieties show diverse quality changes during storage. Studying the spectral changes during this period can assist in determining the optimal storage conditions, extending the shelf-life of apples, reducing losses, and enhancing economic benefits. Moreover, the SSC and fruit firmness directly affect the taste and flavor of apples, which are crucial factors for consumers’ choices. By analyzing the spectral changes, it is possible to predict the market acceptance of apples before they reach maturity, enabling producers to develop more effective marketing strategies.

Current research on fruit primarily focuses on the effects of different storage times or examines individual fruits, frequently considering only a single quality indicator. For instance, Chapanya et al. [3] established a prediction model for syrup stored at various temperatures; predicted values of all three scanned spectra (25, 35, and 45 °C) were not found to be statistically different from the reference value (*p* > 0.05) when using the 25 °C models and combined models for all parameters, providing insights into temperature effects on fruit quality. Guo et al. [4] stored 396 Fuji apples in three conditions (4 °C, 18 °C, and 25 °C) and developed a quality model, discovering that near-infrared spectroscopy combined with a suitable variable selection method shows potential for rapid detection of post-harvest quality, aligning with our research goals. Liu et al. [5] demonstrated that near-infrared (NIR) spectroscopy can be applied for nondestructive detection of SSC, firmness, and moisture content (MC) of pears during maturation, supporting our methodological approach. Ignat et al. [6] used VIS-NIR and SWIR spectrophotometers to detect apple varieties such as ‘Granny Smith’, ‘Pink Lady’, and ‘Starking’ and achieved good results in predicting parameters like total SSC and starch content. This provided a methodological reference and research idea for our study on analyzing the internal quality changes in apples using near-infrared spectroscopy technology. Radenkovs et al. [7] explored the comprehensive effects of various storage techniques on fruit quality, highlighting the importance of optimizing storage methods. From the literature, we determined that it is crucial to investigate the reasons for quality variations in different apple varieties under various storage methods from May to June, which is significant for ensuring quality at the sales end.

Near-infrared spectroscopy combined with chemometric methods has emerged as a reliable and rapid method for evaluating internal quality, widely used for non-destructive detection of fruit quality in recent years [8,9,10]. In this context, spectral preprocessing and band selection methods play a vital role. For example, Du et al. [11] optimized various preprocessing techniques, such as multiple scatter correction and normalization, to successfully identify adulterated low-cost oil types. This highlights the importance of preprocessing in enhancing spectral analysis. Similarly, Wang et al. [12] utilized uninformative variable elimination (UVE) to assess the canopy nitrogen content (CNC) and canopy carbon content (CCC) in maize, demonstrating that UVE-PLS accurately reflects the nitrogen and carbon status of leaves, reinforcing the efficacy of chemometric methods in quality assessment. Liu et al. [13] collected spectra and analyzed the soluble solid content, firmness, and weight of peaches under two storage conditions, establishing PLS models using various preprocessing methods and band selection techniques like UVE and CARS. This research provides valuable insights into internal quality changes during storage. Furthermore, Fan et al. [14] investigated apples with biological variation from 2012 to 2018, focusing on the long-term predictive performance of NIR calibration models for the soluble solid content. They selected CARS and SPA methods to determine the optimal wavelength for predicting apple SSC, achieving an Rp of 0.919 and an RMSEP of 0.592, which showcases the potential of advanced methods in enhancing prediction accuracy.

This study collected spectral data from different apple varieties under various storage methods, along with measurements of their SSC and firmness. We analyze how these two key quality indicators change over different storage durations. Additionally, we employ PLS-DA to classify apples based on their storage time, creating predictive models for SSC and firmness under different storage conditions. By integrating chemometric methods, we aim to optimize these models to enhance their accuracy and reliability. This comprehensive approach not only contributes to a better understanding of the factors affecting apple quality during storage but also provides valuable insights for growers and retailers into how to improve storage practices and maintain high-quality produce for consumers.

## 2. Materials and Methods

### 2.1. Experimental Design

The apple samples used in this experiment included Xinjiang Akesu, Wafangdian Huangyuanshuai, Shandong Fuji, and Luochuan Fuji, with a total of 384 samples for each type. Each type of sample was randomly divided into three groups, with each group containing eight batches. The first group was stored at 25 °C, the second group at 10 °C, and the third group at 1 °C. One batch was taken out for experimental analysis each week over a duration of 7 weeks, and the first batch of each storage method served as the initial samples for comparison. Aside from the temperature variations, the relative humidity was maintained at 75–90% under the 10 °C and 1 °C refrigeration conditions, and at 60–65% at 25 °C, while all other variables were kept constant.

The four apple varieties (Xinjiang Akesu, Wafangdian Huangyuanshuai, Shandong Fuji, and Luochuan Fuji) share certain similarities in their appearance and growing conditions. They typically exhibit similar characteristics in shape and color, and they all adapt well to the specific climate and soil conditions of certain regions in China. Additionally, these varieties may show comparable trends in quality indicators such as SSC and fruit firmness under the same storage conditions.

Despite these similarities, there are significant differences among the four varieties. Wafangdian Huangyuanshuai has a relatively soft flesh that is prone to rot, making it unsuitable for long-term storage, whereas Xinjiang Akesu, Shandong Fuji, and Luochuan Fuji have higher crispness and a better storage performance. Furthermore, the sugar and acidity levels of each variety differ, impacting their flavor and consumer acceptance. These differences become particularly pronounced during storage, affecting their market performance and consumer preferences. Due to Huangyuanshuai’s inability to be stored long-term at 25 °C, the samples began to rot early in the experiment.

### 2.2. Sample Collection

**Figure 1:**
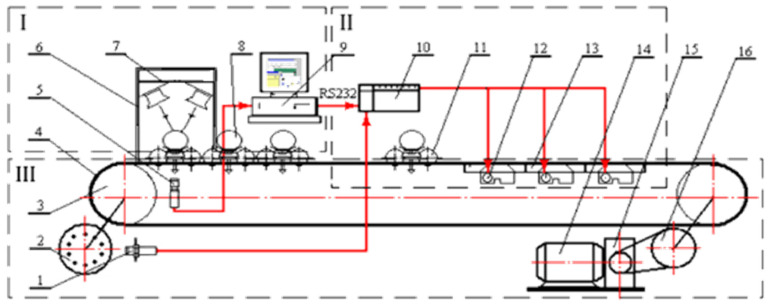
The intelligent online detection equipment for fruit quality. 1. Proximity switch. 2. Encoder disc. 3. Sprocket. 4. Chain. 5. Collimating lens and optical fiber. 6. Dark box. 7. Light source. 8. Sample to be tested. 9. Terminal PC. 10. PLC control cabinet. 11. Fruit tray. 12. Trigger. 13. Grade outlet. 14. Motor. 15. Reducer. 16. Drive sprocket.

This experiment utilized a laboratory-developed intelligent online fruit quality detection device for sample spectral collection, as shown in Figure 1. The QE65Pro spectrometer (Ocean Optics, Orlando, FL, USA) used in this study covers a wavelength range of 350 to 1150 nm. The device operates by activating the PLC control cabinet upon startup.

**Figure 2:**
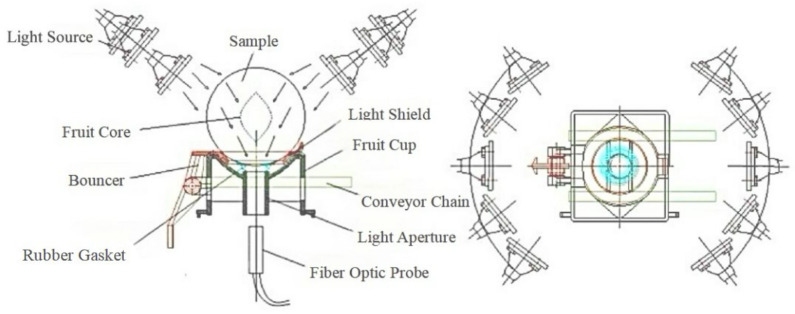
The distribution of diffuse transmitted light sources in the detection equipment.

The experiment employed a diffuse transmission detection method, with Figure 2 illustrating the distribution of the diffuse transmission light source. To enhance measurement accuracy, spectral measurements were taken at 90° intervals around the equator of the sample, and the average of four measurements was used for subsequent analysis. Before collecting the spectral data of the experimental samples, the device should be turned on in advance to achieve a stable light source intensity, ensuring the accuracy of the data. The integration time was set to 100 milliseconds, and dark spectra were removed using the accompanying software. To construct a more reliable and predictable regression model, the Kennard–Stone algorithm was used to randomly divide each sample into calibration and prediction sets in a 3:1 ratio. Specifically, there were 288 samples in the calibration set and 96 samples in the prediction set. Please refer to the Supplementary Materials for device details.

### 2.3. Measurement of Physicochemical Indicators

Upon completion of spectral collection, the firmness measurement of the apples was carried out using an FTC texture analyzer (Food Technology Corporation, Sterling, VA, USA). This instrument was equipped with a 3 mm cylindrical probe, which was specifically designed to ensure accurate and consistent measurements. Before the test, each experimental apple had its equatorial part marked at 90° intervals. After spectral collection, the marked positions on the apples were carefully aligned and placed in close contact with the cylindrical probe. During the test, the probe was precisely controlled to penetrate the apple. The instrument’s accompanying software played a crucial role in data processing. It was programmed to record and analyze the force data generated during the probe’s penetration. As the probe exerted pressure on the apple, the firmness data were instantaneously transmitted to the computer connected to the instrument. By collecting and analyzing these data points, we were able to obtain the firmness values for each sample accurately.

Following the collection of firmness data, the SSC of apple samples was immediately measured using a PAL-1 temperature-compensated refractometer manufactured by ATAGO, Tokyo City, Japan. First, the refractometer was calibrated with distilled water. Then, a manual juicer was used to extract the juice, which was introduced to the center of the refractometer prism for optical measurement. After each measurement, the prism was washed with distilled water to prevent cross-contamination. Additionally, to improve data reliability, each sample was measured in triplicate, and the average value was recorded as the final result.

### 2.4. Data Processing and Chemometric Methods

#### 2.4.1. Preprocessing and Variable Selection

In this study, the use of multiple signal-processing techniques is based on several considerations. Although near-infrared spectroscopy can obtain information about the internal quality of apples, the spectral data are complex, containing a large amount of information and noise that is irrelevant to the internal quality. A single signal-processing technique struggles to comprehensively remove these interfering factors. In this experiment, three commonly used spectral preprocessing methods were employed to enhance the validity and reliability of the data: MSC, SNV, and normalization. MSC is particularly effective in removing scatter effects that arise from variations in sample presentation and instrument response, thus providing a more standardized dataset for analysis. SNV transforms the spectral data by centering and scaling them, which helps to address multiplicative effects and enhances comparability across samples. Normalization further ensures that the data remain within a consistent range, reducing the impact of sample concentration and path length variations.

Additionally, for band selection, the CARS [15,16] algorithm and UVE [17] method were applied for effective comparison and analysis. CARS is advantageous as it not only selects informative wavelengths but also reduces the dimensionality of the dataset, thereby improving model interpretability. UVE, on the other hand, systematically identifies and eliminates non-informative variables, further refining the dataset to focus on the most relevant spectral features. The integration of these techniques is innovative in apple storage quality research. For instance, standardization and MSC first optimize the spectral baseline and reduce scattering effects, and then SNV further normalizes the data. CARS and UVE are then applied to select the most relevant wavelengths. This step-by-step process allows for more accurate extraction of spectral information related to apple internal quality, which is different from previous studies that usually use only one or two of these methods.

Through these preprocessing steps, the aim is to minimize the influence of noise on the results, thereby improving the performance and accuracy of the model. By ensuring that only the most pertinent data are utilized for analysis, we enhance the robustness of our multivariate models, ultimately leading to more reliable predictions and insights in the context of this study.

#### 2.4.2. Modeling Methodology

Partial least squares regression (PLSR) is a chemometric technique that connects the spectral data of fruits with quality levels. It can effectively address situations where the input variables contain noise and are highly correlated. Over the past few decades, it has been widely applied in the modeling of fruits and vegetables. The principle of PLSR involves decomposing the spectral matrix X to eliminate irrelevant noise while also considering the influence of the concentration matrix Y. PLSR is a multivariate factor regression method based on this principle. In this study, the PLSR algorithm was applied to establish models for predicting the SSC value and firmness of apples. When Y comprises categorical data, PLSR is referred to as PLS-DA. PLS-DA is employed for classification in this study for several compelling reasons. Firstly, it is a powerful statistical approach adept at managing high-dimensional data and precisely differentiating among distinct sample classes. In the context of this research, it classifies apple samples effectively based on spectral data corresponding to various storage durations, which is crucial for identifying optimal storage conditions. Secondly, sample size significantly impacts the PLS-DA model. Generally, as the sample size grows, the model’s stability and reliability improve. A larger number of samples offers a more comprehensive feature set, enabling the model to better learn and capture data patterns. Conversely, an insufficient sample size may result in overfitting, thereby undermining the classification performance. Finally, PLS-DA has practical applications in real-time apple quality monitoring. It can assist producers in determining the optimal sales timing by analyzing spectral data under different storage conditions and provide specific storage recommendations to growers by obtaining the best latent variable (LV) using cross-validation of the modeling set. The model’s performance was evaluated using parameters such as the RMSEP, RMSEC, Rp, and Rc. It should be noted that the unit of RMSEP depends on the unit of the predicted variable. In this study, for the prediction of SSC of apples, the unit of SSC is °Brix, so the unit of RMSEP for SSC prediction models is also °Brix. For fruit firmness, which is measured in Newtons (N), the unit of RMSEP for firmness prediction models is also Newtons (N). These units help to directly reflect the average error between the predicted and actual values of the corresponding variables, providing a clear indication of the model’s prediction accuracy.

## 3. Analysis and Discussion

### 3.1. Sample Characterization

**Figure 3:**
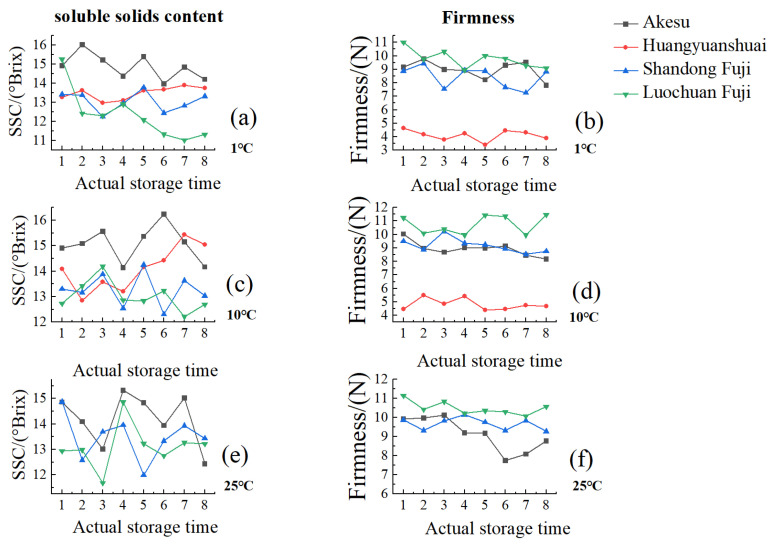
Changes in SSC and firmness of apples of different varieties under different storage conditions. (**a**) Changes in SSC of different experimental varieties at 1 °C; (**b**) Changes in firmness of different experimental varieties at 1 °C; (**c**) Changes in SSC of different experimental varieties at 10 °C; (**d**) Changes in firmness of different experimental varieties at 10 °C; (**e**) Changes in SSC of different experimental varieties at 25 °C; (**f**) Changes in firmness of different experimental varieties at 25 °C.

In Figure 3, the average weekly variations in sweetness and firmness across different apple varieties under various storage conditions are presented. Table 1 illustrates the standard deviations and mean values of the SSC and firmness of different apple varieties under different storage conditions.

Notably, under storage temperatures of 1 °C and 10 °C, the firmness of Wafangdian Huangyuanshuai is observed to be lower than that of Akesu, Shandong Fuji, and Luochuan Fuji. This disparity is attributed to Huangyuanshuai’s mealy texture, in contrast to the firmer and crisper flesh of Akesu, Shandong Fuji, and Luochuan Fuji. The crispness of apple flesh is primarily influenced by the levels of insoluble pectin and cellulose present in the cellular structure. Consequently, Huangyuanshuai exhibits relatively low firmness and is prone to spoilage within 5–6 days when stored at room temperature.

Under 1 °C and 10 °C, the sweetness and firmness of Akesu, Shandong Fuji, and Luochuan Fuji demonstrate a gradual decline within a certain range, a process that occurs at a slower rate than at 25 °C, where respiration initially increases before declining. This observation supports the findings of Radenkovs et al. [7], who highlighted the importance of temperature in regulating respiration rates and moisture loss in fruit. During storage, refrigeration effectively suppresses the respiration rate of apples and minimizes moisture loss. Conversely, at room temperature, respiration is not inhibited, resulting in rapid moisture evaporation. Due to the characteristics of its flesh, Huangyuanshuai experiences an oscillating increase in sweetness, while its firmness varies only slightly as moisture content decreases.

Throughout all three storage conditions, Shandong Fuji consistently exhibits higher sweetness and lower firmness compared to Luochuan Fuji. Overall, Akesu shows more pronounced fluctuations in quality, whereas Fuji apples maintain a relatively stable quality during storage; Huangyuanshuai, however, demonstrates the least favorable performance.

### 3.2. Spectral Characterization

**Figure 4:**
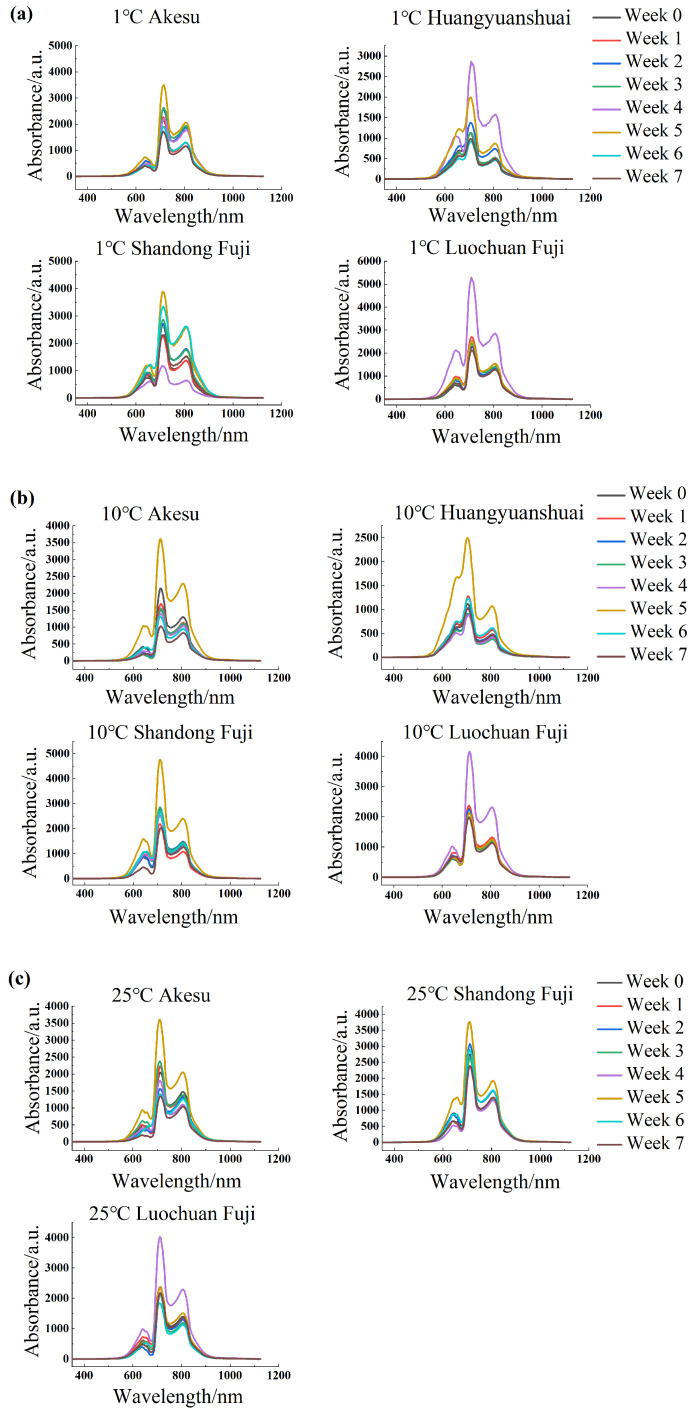
Average spectral difference in different apple varieties under different storage methods. (**a**) Average spectral change during storage at 1 °C; (**b**) average spectral change during storage at 10 °C; (**c**) average spectral variation at 25 °C.

The average spectra of various apple cultivars under different storage conditions are illustrated in Figure 4. The spectra were recorded over a wavelength range of 350 to 1150 nm, encompassing a total of 1044 variables. Notably, the spectrum of Huangyuanshuai exhibits considerable variation within the 630–675 nm range. This variation is primarily attributed to alterations in carotenoids and phenolic compounds in the skin. The variation in these pigments is related to the suppression of respiration during storage, resulting in a rightward shift of the spectral peaks and an increase in intensity.

The peak observed around 720 nm is primarily attributed to the overtone stretching vibrations of C-H and O-H bonds, which are indicative of the presence of water and organic compounds in the apple flesh. This peak is particularly relevant as it reflects changes in the internal quality of the apples during storage. When the water content is sufficient, the intensity of this absorption peak is relatively high, suggesting a firmer and crisper texture. However, as moisture loss occurs—especially in samples stored at 10 °C and 25 °C—this peak weakens, indicating a decline in internal quality.

Samples stored at 1 °C demonstrated optimal preservation, exhibiting less moisture loss compared to those maintained at 10 °C and 25 °C. Initially, the spectral peaks of these samples were relatively high. However, as storage time increased, the moisture loss in samples at 10 °C and 25 °C also increased, leading to a weakening of the O-H bond absorption vibrations at 720 nm. This weakening caused a decrease in the intensity of this absorption peak, which enhances light transmittance within the fruit.

As a result, the relative intensity of other peaks in the spectrum gradually increased. This indicates that both storage temperature and duration significantly impact the recorded spectra, with effects being more pronounced at 10 °C and 25 °C, particularly in relation to the changes in moisture content and internal quality. Additionally, the internal characteristics of the apples, such as sugar and acidity, also influence these vibrational modes. An increase in sugar content typically enhances the C-H bond vibrations, further affecting the overtone absorption peaks. Furthermore, the cellulose content in the flesh is closely related to the fruit’s crispness; variations in cellulose impact the structural integrity of the flesh, consequently affecting the intensity and position of the overtones.

Interestingly, Małachowska [18] emphasized the importance of maintaining firmness in the Gala Schniga SchniCo Red(s) cultivar during storage and transport. The study emphasized that the use of 1-MCP and optimal harvest dates significantly affected fruit quality. For instance, apples treated with Harvista™ maintained firmness above 55 N during simulated transport conditions (lower storage temperature) when harvested at the optimal time. This aligns with our findings, as maintaining a lower storage temperature (1 °C) and managing moisture loss are crucial for preserving internal quality and firmness in apples.

Thus, the analysis of these spectral characteristics provides valuable insights into the quality changes in apples during storage, enabling better management of storage conditions to maintain fruit quality.

### 3.3. Comparison of Discriminant Models for Apples

**Figure 5:**
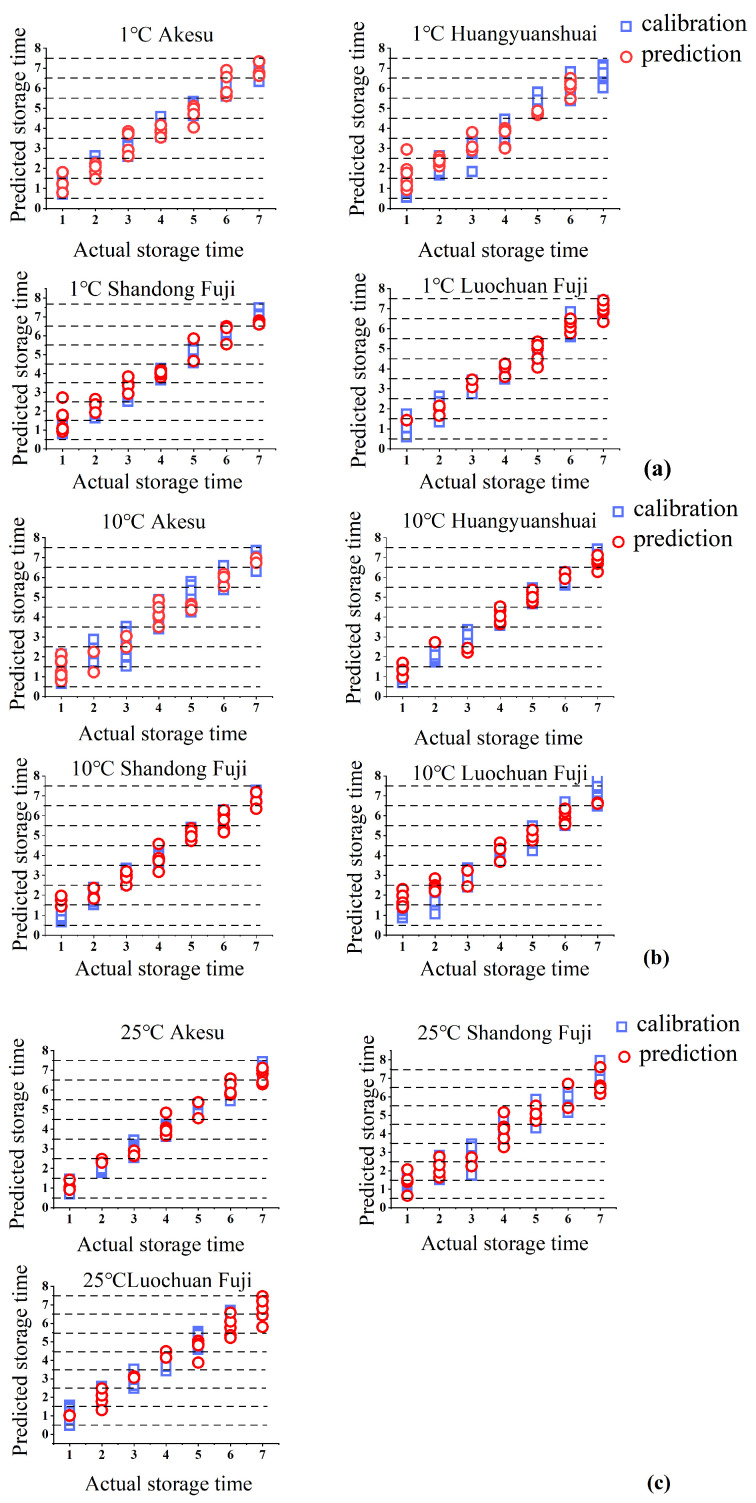
Partial least squares discriminant model of different apple varieties under different storage temperatures. (**a**) Discriminant model for storage period at 1 °C; (**b**) discriminant model for storage period at 10 °C; (**c**) discriminant model for storage period at 25 °C.

In this study, PLS-DA was employed to develop discriminant models for sample storage durations across various storage conditions, as illustrated in Figure 5. The storage weeks 1 through 7 were designated as the true discriminant values, with the midpoint acting as the boundary. Variations in respiration duration lead to changes in soluble solids and firmness within the fruit, resulting in distinct differences in the apple spectra, which facilitate effective discrimination among the samples.

As presented in Table 2, the PLS-DA model for Shandong Fuji apples stored at 1 °C exhibited exceptional discriminative capability, achieving a misclassification rate of 0% in the prediction set. This outstanding performance is attributed to marked changes in sweetness, reflecting an expansion of internal quality disparities. Conversely, the model for Akesu apples stored at 10 °C demonstrated suboptimal performance, with nine misclassifications in the prediction set and a misclassification rate of 32.1%. This can be explained by the suppression of respiration at 10 °C, which resulted in relatively minor spectral alterations and diminished discrimination efficacy.

It is evident that a poorer model performance was associated with refrigerated conditions. The suppression of respiration during refrigeration leads to smaller internal quality changes, thereby reducing the differences between samples. At 25 °C, the discriminant results for the three apple varieties were consistent. This consistency arises from the fact that, under identical conditions, the internal quality differences among the various apple varieties were obscured by vigorous respiration, resulting in similar discrimination outcomes.

Zhang et al. [19] utilized near-infrared spectroscopy to develop models for SSC and dry matter content (DMC) across eight apple varieties. Their findings indicated that models created for individual apple varieties exhibited higher prediction accuracy. However, reliance on data from single varieties may lead to overfitting, and the distribution of actual values (SSC and DMC) could negatively impact predictions during external validation. Figure 5, which encompasses multiple varieties, shows comparable or even better performance in some aspects, suggesting that our multi-variety approach can address the overfitting issue pointed out by Zhang et al. Consequently, to achieve robust predictive outcomes, it is essential to construct models that encompass multiple varieties. This underscores the necessity of a multi-variety storage-period model for accurate predictions, rather than depending solely on single-variety models.

### 3.4. Construction of the Predictive Model

#### 3.4.1. Spectral Preprocessing

**Figure 6:**
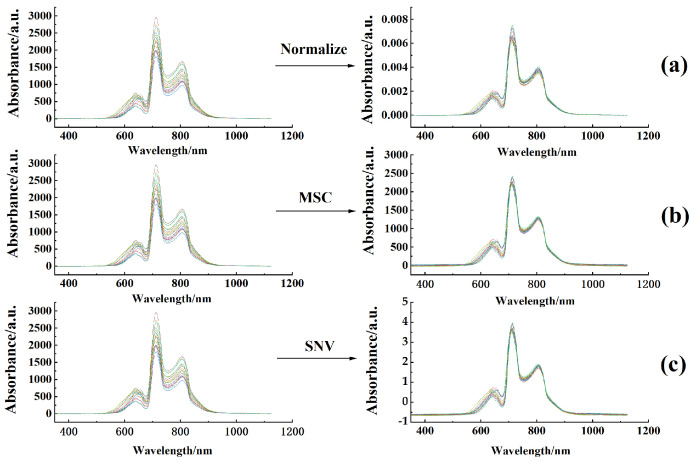
Spectral pretreatment effect. (**a**) The original spectra were normalized; (**b**) MSC was applied to the original spectra; (**c**) SNV was applied to the original spectra.

This study employed PLS regression to develop predictive models for sweetness and firmness, configuring the number of LVs at between 1 and 20 to mitigate the risks of overfitting and underfitting. As illustrated in Figure 6a, the original spectra were normalized to correct for spectral variations arising from slight differences in path length, and the correlation coefficients were subsequently optimized. In Figure 6b, MSC was applied to the original spectra, effectively removing linear variations due to scattering. This process resulted in a reduced number of LVs and enhanced correlation coefficients. Figure 6c depicts the SNV transformation, which successfully mitigated the influences of solid particle size, surface scattering, and variations in path length on diffuse reflectance, thereby yielding superior results compared to the original spectra. Following spectral preprocessing, pre-modeling assessments revealed that the normalized spectra produced the most favorable preprocessing outcomes. Nevertheless, the firmness model derived from the SNV-processed original spectra at 25 °C outperformed that based on the normalized data. The optimized spectra are presented in Figure 6.

#### 3.4.2. SSC and Firmness Modeling

Before building the model, the full-spectrum data contain a large amount of redundant information. Using all of them will not only increase the model’s computational load but also easily introduce redundant information, leading to overfitting. The UVE and CARS band selection methods can effectively extract relevant information and improve model quality. These methods simplify the data structure by eliminating irrelevant variables and selecting the most informative features, which enhances the model’s generalization ability. The CARS algorithm can effectively select the wavelength variables most valuable for predicting the internal quality of apples through an adaptive reweighting mechanism, reducing the data dimensionality and improving the model’s interpretability. UVE, on the other hand, systematically identifies and removes variables with minimal impact on the prediction results, streamlining the dataset and allowing the model to focus more on key information, thus enhancing the accuracy and stability of the model’s predictions.

**Figure 7:**
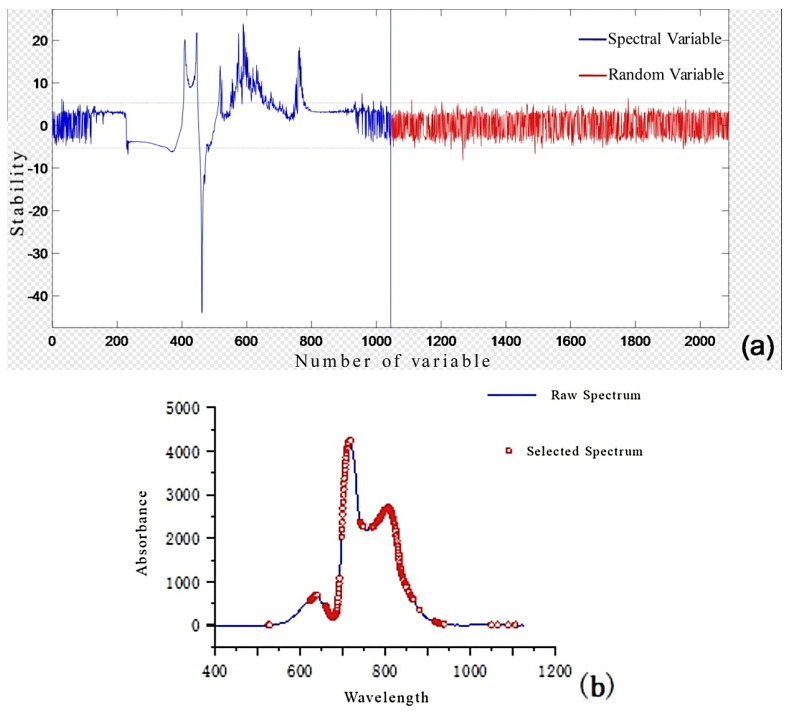
Results of UVE wavelength selection (**a**) selection process; (**b**) selected variables.

The number of random variables used in UVE was set to 1044, aligning with the spectral variables. As shown in Figure 7, the values of the top and bottom dashed lines represent the threshold. Spectral variables within the threshold were eliminated, while those outside the threshold were considered effective variables. The selected spectral variables included a large number of peak and valley variables.

**Figure 8:**
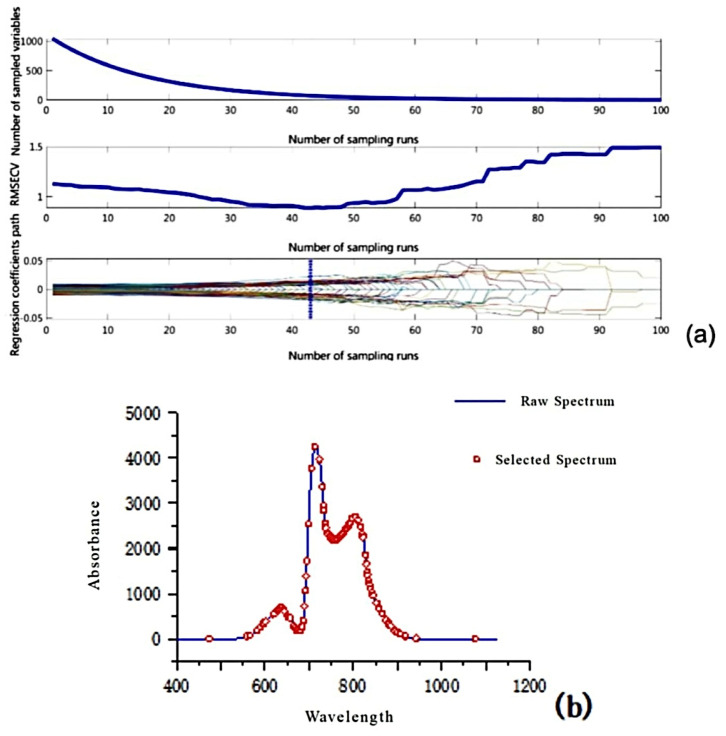
Results of CARS wavelength selection: (**a**) selection process; (**b**) selected variables.

The CARS algorithm was employed to select the spectral parameters of the samples. Figure 8 illustrates the results of this selection process, using 100 Monte Carlo samples and 10-fold cross-validation. It is evident that in the first 43 samples, the RMSECV values decrease, which indicates that variables not related to the internal quality of the samples are being eliminated, corroborating the results of Guo et al. [4]. However, after the first 43 samples, the RMSECV values continue to rise, suggesting that variables associated with the internal quality of the samples are gradually being removed during the selection process.

**Figure 9:**
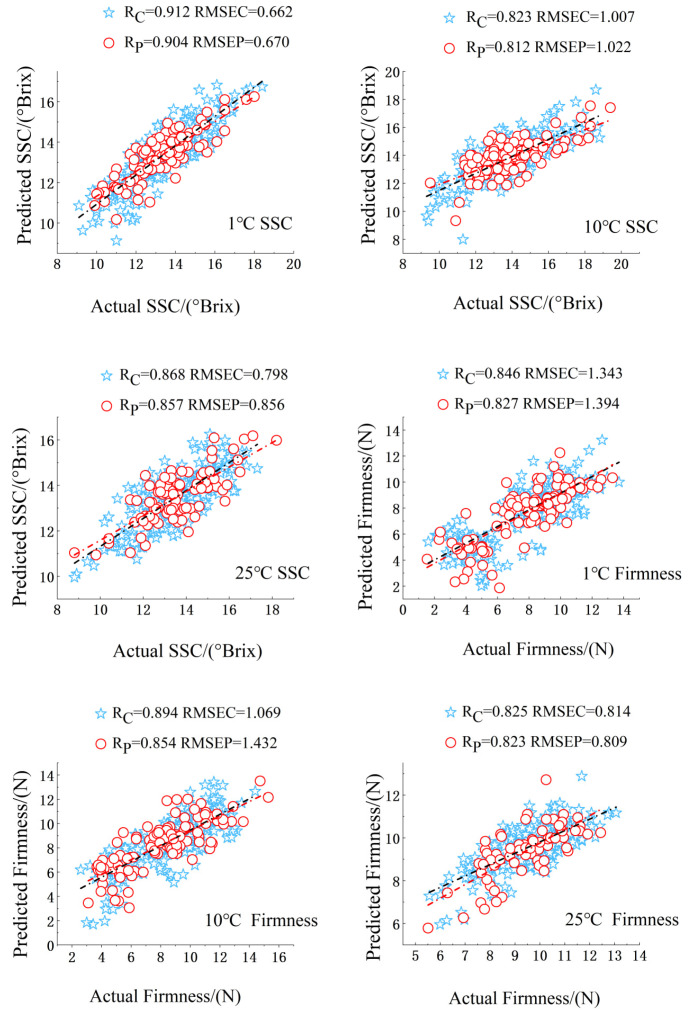
Optimal prediction model of samples under different storage periods.

Table 3 presents a comparison of the effects of models developed using the original spectra following preprocessing and variable selection against those constructed from the unprocessed original spectra. It is evident that both the CARS and UVE algorithms effectively reduce the number of spectral variables; however, CARS distinctly enhances model performance, diminishes dimensionality, and elevates correlation coefficients, whereas UVE shows comparatively less efficacy. The integration of preprocessing with variable selection algorithms results in superior outcomes. From Table 3, it is apparent that the Rp and RMSEP values for the samples significantly improved after applying spectral variable selection via CARS. The models demonstrated a strong capacity to predict the SSC and firmness of the samples. For the optimal model at 1 °C, the Rp values for SSC and firmness were recorded at 0.904 and 0.827, with RMSEP values of 0.670 and 1.394, respectively. At 10 °C, the optimal model yielded Rp values of 0.812 and 0.854 for SSC and firmness, with corresponding RMSEP values of 1.022 and 1.432. For the optimal model at 25 °C, the Rp values for SSC and firmness were 0.857 and 0.823, with RMSEP values of 0.856 and 0.809, respectively. The models proved effective in predicting the SSC and firmness of the samples. Figure 9 illustrates the optimal prediction models for apples under various storage conditions. When constructing the model, the cross-validation method was used to determine the optimal number of LVs. Through multiple cross-validations, the performance of the model can be more accurately evaluated, and the model’s overreliance on a certain portion of the data can be avoided. Moreover, the model performance was not judged solely based on the two indicators, RMSEP and RMSECV. Instead, multiple indicators, such as Rp and Rc, were combined for a comprehensive evaluation of the model. Judging from the comprehensive evaluation results, the model performs well in predicting the sugar content and firmness of apples, indicating that the model has good generalization ability and is not the result of overfitting.

Bo et al. [20] advanced predictive models for SSC and firmness throughout the cold storage period across three apple maturity stages, employing the non-destructive detection capabilities of near-infrared spectroscopy. The correlation coefficient (Rp) for SSC varied from 0.86 to 0.94, while the Rp for firmness ranged between 0.82 and 0.86. As depicted in Table 3, our Rp values for SSC and firmness fall within a similar range, confirming that the non-destructive near-infrared spectroscopy approach, in combination with appropriate variable selection and preprocessing, can lead to reliable predictive models. These results affirm that the predictive models for SSC and firmness of apples, established through near-infrared spectroscopy in conjunction with partial least squares (PLS), are highly reliable, indicating the potential for further optimization of the models developed in this study. Our findings, in conjunction with prior research, suggest that the application of non-destructive near-infrared spectroscopy for predicting SSC and firmness during the storage period of apples is viable, offering an efficient and practical methodology for these predictions.

## 4. Conclusions

This study utilizes intelligent online detection technology to assess fruit quality, leveraging diffuse transmission to acquire spectra from various apple varieties under differing storage conditions, while measuring their SSC and firmness. As the storage duration extends, the flesh of the apples progressively softens, resulting in a reduction in firmness, whereas SSC fluctuates within a defined range. However, the decline in firmness is mitigated as storage temperature decreases, due to the suppression of respiration, which facilitates the conversion of flesh to pectin and decelerates moisture loss. The transition from 10 °C to 1 °C is particularly pronounced, primarily because respiration is significantly inhibited at these lower temperatures. The spectral data reveal a peak in respiration intensity occurring between the third and fifth weeks of storage, after which the intensity begins to decline.

Furthermore, this study employs PLS pre-modeling, filtering out anomalies using the Shapiro–Wilk criterion, and establishes a discriminative model via PLS-DA. The model demonstrates a low misclassification rate and exhibits strong performance, attributed to the respiratory changes in apples as storage days progress, which are reflected in the increased variability of spectral information among individual samples. It was determined that the optimal storage duration for apples is between 3 and 5 weeks within a 7-week storage period prior to maturity. The Huangyuanshuai variety is unsuitable for prolonged storage at 25 °C after extended periods. As the storage temperature decreases, the internal quality changes in the apples gradually stabilize. Overall, the Akesu variety displays relatively large fluctuations in internal quality during storage, whereas the Shandong Fuji and Luochuan Fuji varieties exhibit more stable characteristics.

Additionally, various preprocessing techniques were employed in conjunction with the UVE and CARS variable selection algorithms to establish predictive models for the SSC and firmness of apple samples. Comparative analysis indicated that the model derived from normalization combined with CARS variable selection yielded the most favorable results. The normalization-CARS-PLS model for SSC at 1 °C achieved the optimal performance, with an Rp of 0.904 and an RMSEP of 0.67. For firmness at an ambient temperature, the normalization-CARS-PLS model also demonstrated a superior performance, achieving an Rp of 0.823 and an RMSEP of 0.809. This research successfully established discriminative models for four apple varieties stored under three different conditions for varying durations, and it developed predictive models for SSC and firmness at three storage temperatures, all attaining commendable predictive results. The intelligent online detection technology and modeling approach proposed in this study offer a promising framework that could potentially be extended to assess the quality of other fruit species and cultivars. This would not only enhance the understanding of fruit quality changes during storage but also contribute to optimizing storage conditions across a broader range of fruits, providing valuable guidance for the fruit storage industry.

## Figures and Tables

**Table 1:** The standard deviations and mean values of the SSC and firmness of different apple varieties under different storage conditions.

Storage Temperature	Variety	Standard Deviation of Soluble Solid Content	Mean Value of Soluble Solid Content	Standard Deviation of Firmness	Mean Value of Firmness
1 °C	Akesu	0.676	14.874	0.660	8.970
	Huangyuanshuai	0.333	13.499	0.396	4.119
	Shandong Fuji	0.524	13.055	0.805	8.434
	Luochuan Fuji	1.345	12.338	0.681	9.781
10 °C	Akesu	0.698	15.078	0.550	8.933
	Huangyuanshuai	0.878	14.100	0.426	4.815
	Shandong Fuji	0.653	13.263	0.532	9.178
	Luochuan Fuji	0.591	13.020	0.694	10.731
25 °C	Akesu	1.027	14.188	0.886	9.130
	Shandong Fuji	0.884	13.474	0.324	9.676
	Luochuan Fuji	0.873	13.116	0.352	10.493

**Table 2:** Results of the discriminant model.

Storage Temperature	Variety	Misclassification Rate of the Calibration Set/100%	Misclassification Rate of the Validation Set/100%
1 °C	Akesu	0.060	0.214
	Huangyuanshuai	0.131	0.214
	Shandong Fuji	0	0.143
	Luochuan Fuji	0.071	0
10 °C	Akesu	0.202	0.321
	Huangyuanshuai	0	0.071
	Shandong Fuji	0.048	0.214
	Luochuan Fuji	0.142	0.250
25 °C	Akesu	0.060	0.214
	Shandong Fuji	0.060	0.214
	Luochuan Fuji	0.060	0.214

**Table 3:** Effect of wavelength selection algorithm model.

Quality Parameters	Storage Temperature	Treatment Method	Latent Variables	RMSEC	$R_{C}$	RMSEP	$R_{P}$
SSC	1 °C	Raw spectra	13	0.846	0.851	0.873	0.834
		Normalization	15	0.632	0.92	0.819	0.855
		MSC	14	0.701	0.901	0.844	0.845
		SNV	15	0.687	0.904	0.837	0.848
		Normalization-cars	13	0.662	0.912	0.670	0.904
		Normalization-uve	11	0.957	0.805	1.076	0.735
	10 °C	Raw spectra	12	1.112	0.779	1.226	0.710
		Normalization	13	0.986	0.831	1.232	0.706
		MSC	12	1.01	0.82	1.227	0.710
		SNV	13	1.02	0.819	1.228	0.710
		Normalization-cars	12	1.007	0.823	1.022	0.812
		Normalization-uve	9	1.163	0.755	1.426	0.580
	25 °C	Raw spectra	11	0.999	0.784	1.067	0.760
		Normalization	12	0.895	0.831	1.025	0.786
		MSC	11	0.926	0.818	1.029	0.781
		SNV	12	0.915	0.823	1.030	0.781
		Normalization-cars	11	0.798	0.868	0.856	0.857
		Normalization-uve	6	1.316	0.575	1.406	0.513
Firmness	1 °C	Raw spectra	10	1.524	0.801	1.535	0.788
		Normalization	10	1.408	8.33	1.470	0.808
		MSC	9	1.482	0.813	1.486	0.801
		SNV	10	1.416	0.831	1.492	0.798
		Normalization-cars	7	1.343	0.846	1.394	0.827
		Normalization-uve	8	1.511	0.805	1.43	0.816
	10 °C	Raw spectra	11	1.444	0.804	1.69	0.792
		Normalization	9	1.376	0.824	1.648	0.801
		MSC	9	1.383	0.822	1.641	0.802
		SNV	9	1.453	0.801	1.625	0.805
		Normalization-cars	12	1.069	0.894	1.432	0.854
		Normalization-uve	8	1.55	0.77	1.704	0.784
	25 °C	Raw spectra	10	0.986	0.728	0.992	0.727
		Normalization	10	0.983	0.73	1.014	0.698
		MSC	10	0.982	0.731	1.005	0.703
		SNV	11	0.94	0.757	0.996	0.710
		SNV-cars	9	0.814	0.825	0.809	0.823
		SNV-uve	7	1.117	0.631	1.032	0.683

## Data Availability

The original contributions presented in this study are included in the article/Supplementary Materials. Further inquiries can be directed to the corresponding author.

## Supplementary Materials

The following supporting information can be downloaded at: https://www.mdpi.com/article/10.3390/foods14081412/s1, Figure S1: The intelligent online detection equipment for fruit quality; Figure S2: The distribution of diffuse transmitted light sources in the detection equipment.
