# Supplementary material for: Analysis of Internal Quality Changes in Apples During Storage Using Near-Infrared Spectroscopy
Source: Foods. 2025 Apr 18;14(8):1412. doi: 10.3390/foods14081412 (PMC12027357; doi:10.3390/foods14081412)
Supplement: Supplementary file 1 [file foods-14-01412-s001.zip › foods-3554689-supplementary.pdf]

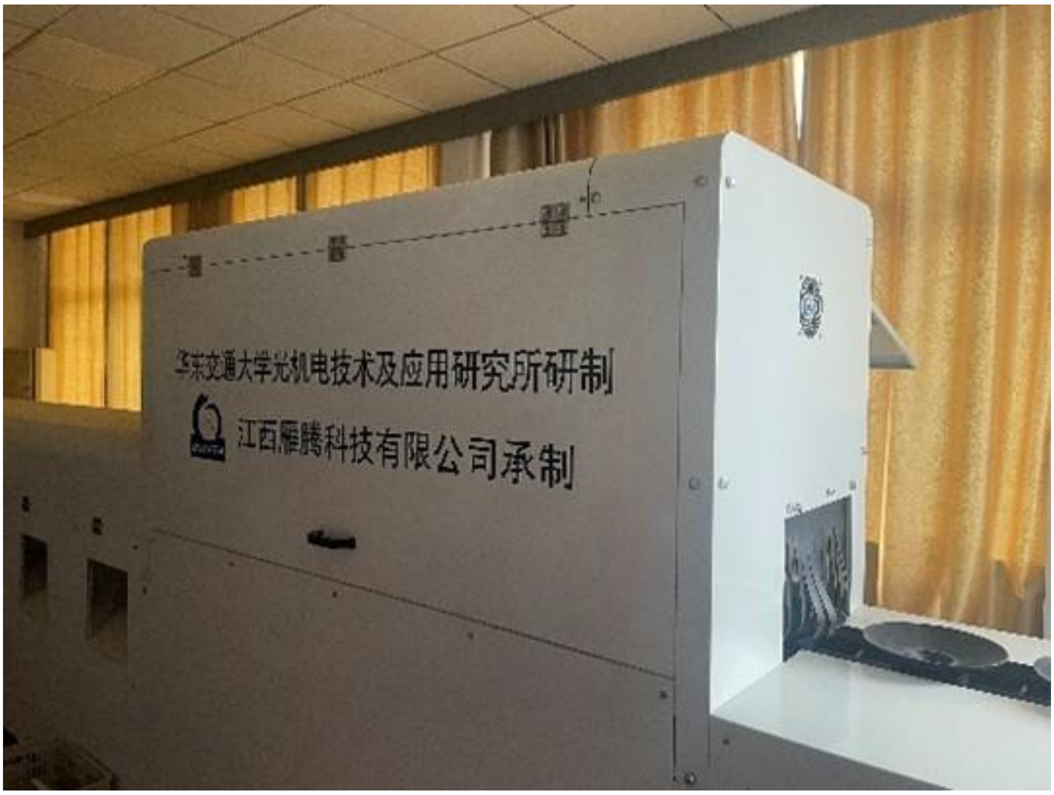

Figure 1. The intelligent online detection equipment for fruit quality.

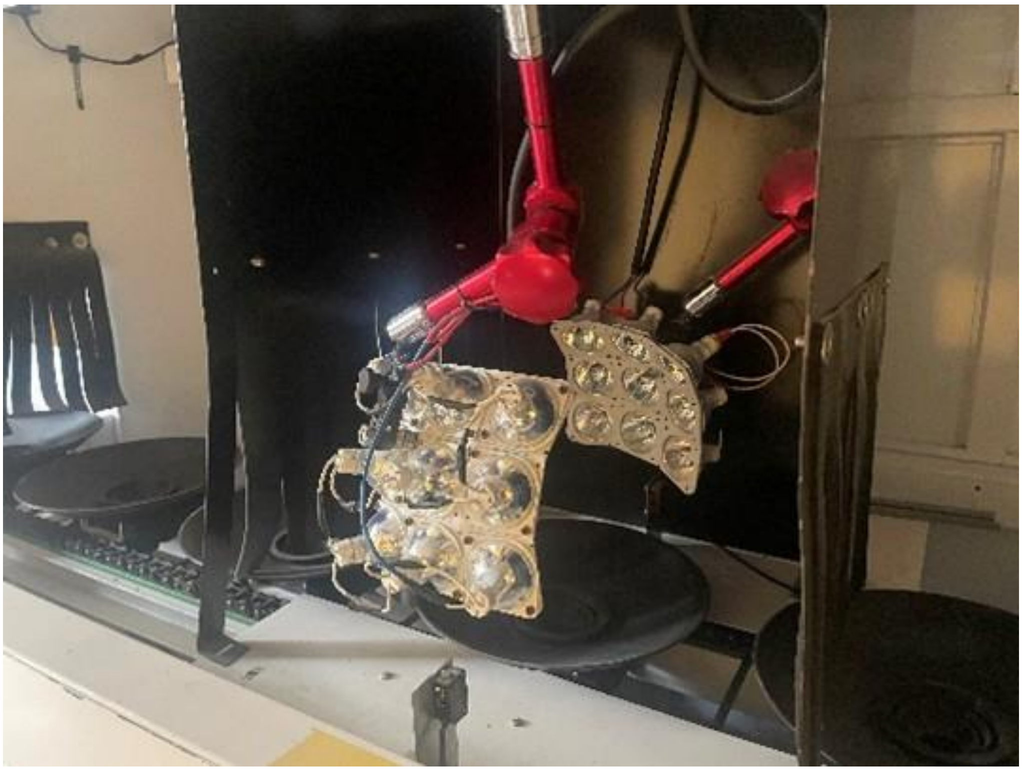

Figure 2. The distribution of diffuse transmitted light sources in the detection equipment.
